# Resibufogenin Induces G1-Phase Arrest through the Proteasomal Degradation of Cyclin D1 in Human Malignant Tumor Cells

**DOI:** 10.1371/journal.pone.0129851

**Published:** 2015-06-29

**Authors:** Masami Ichikawa, Yoshihiro Sowa, Yosuke Iizumi, Yuichi Aono, Toshiyuki Sakai

**Affiliations:** Department of Molecular-Targeting Cancer Prevention, Graduate School of Medical Science, Kyoto Prefectural University of Medicine, Kyoto, Japan; Virginia Commonwealth University, UNITED STATES

## Abstract

Huachansu, a traditional Chinese medicine prepared from the dried toad skin, has been used in clinical studies for various cancers in China. Resibufogenin is a component of huachansu and classified as bufadienolides. Resibufogenin has been shown to exhibit the anti-proliferative effect against cancer cells. However, the molecular mechanism of resibufogenin remains unknown. Here we report that resibufogenin induces G1-phase arrest with hypophosphorylation of retinoblastoma (RB) protein and down-regulation of cyclin D1 expression in human colon cancer HT-29 cells. Since the down-regulation of cyclin D1 was completely blocked by a proteasome inhibitor MG132, the suppression of cyclin D1 expression by resibufogenin was considered to be in a proteasome-dependent manner. It is known that glycogen synthase kinase-3β (GSK-3β) induces the proteasomal degradation of cyclin D1. The addition of GSK-3β inhibitor SB216763 inhibited the reduction of cyclin D1 caused by resibufogenin. These effects on cyclin D1 by resibufogenin were also observed in human lung cancer A549 cells. These findings suggest that the anti-proliferative effect of resibufogenin may be attributed to the degradation of cyclin D1 caused by the activation of GSK-3β.

## Introduction

**Fig 1:**
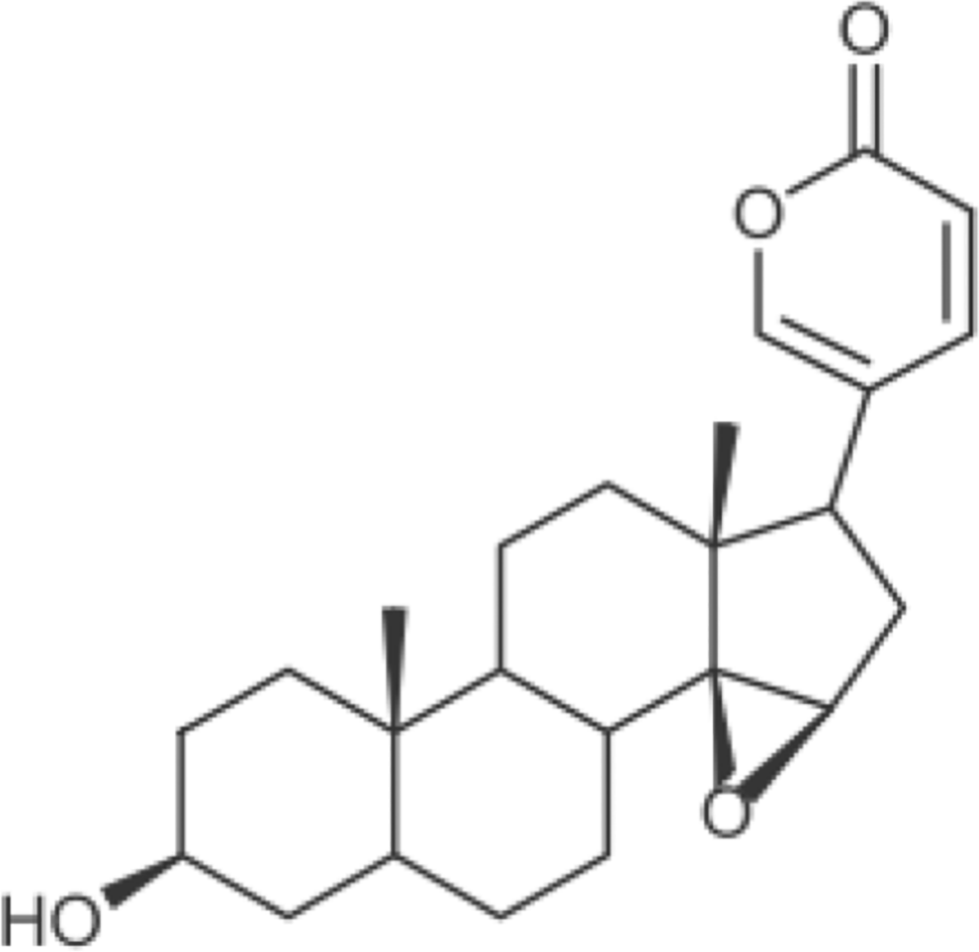
Structural formula of resibufogenin.

Huachansu, a traditional Chinese medicine, is dried venom secreted from the skin glands of *Bufo bufo gargarizans* Cantor [1]. It was reported that huachansu suppresses the growth of human lung cancer H460, A549 and H1299 cells *in vitro* [2]. Moreover, in China, a meta-analysis showed that combined treatment of huachansu with conventional chemotherapeutic agents was more effective in increasing response rate and Karnofsky score than chemotherapeutic agents alone against gastric cancer patients [3]. Huachansu was also used for clinical studies in patients with other advanced malignancies [4–6]. Resibufogenin (Fig 1) is a component of huachansu and has been shown to inhibit the growth of human hepatocellular cancer HepG2 and Bel-7402 cells [7, 8]. Moreover, resibufogenin also inhibited the growth with G2/M-phase arrest in human hepatocellular cancer SMMC-7721 cells [8]. However, precise molecular mechanism of the growth inhibition by resibufogenin is still unknown.

G1 to S-phase transition is regulated by cyclin-dependent kinases (CDKs) 2/4/6 with cyclin D/E [9]. Cyclin D1 and cyclin E activate CDK4/6 and CDK2, respectively. Cyclin D1-CDK4/6 and cyclin E-CDK2 phosphorylate retinoblastoma (RB) protein at Ser^780^ and Ser^807/811^ sites, respectively, and these phosphorylations are required to completely inactivate RB protein [10]. The RB Ser^807/811^ phosphorylation by cyclin E-CDK2 requires RB Ser^780^ phosphorylation by cyclin D1-CDK4/6 [11]. Therefore, cyclin D1 is important in G1 to S-phase transition, and is over-expressed in many human malignant tumors [12–14]. The transcription of cyclin D1 is activated by the accumulation of β-catenin as a result of loss of functional adenomatous polyposis coli protein in colon cancer [15].

The expression of cyclin D1 is regulated by not only transcription but also degradation. The stability of cyclin D1 is regulated by proteasomal degradation [16]. The cyclin D1 degradation is triggered by the phosphorylation [17] and the phosphorylation is caused by glycogen synthase kinase-3β (GSK-3β) [18, 19].

In this study, we elucidated the molecular mechanism of the growth inhibition by resibufogenin using human colon cancer HT-29 cells and human lung cancer A549 cells. We found that resibufogenin induced G1-phase arrest by down-regulation of cyclin D1 protein through the proteasomal degradation resulting in hypophosphorylation of RB protein.

## Materials and Methods

### Cell culture

Human colon cancer HT-29 cell line was purchased as a cell line of the NCI-60 from the NCI Developmental Therapeutics Program. Human lung cancer A549 cell line was purchased from ATCC. HT-29 cells and A549 cells were maintained in DMEM and RPMI-1640, respectively. These media were supplemented with 10% FBS, 4 mM or 2 mM L-glutamine for DMEM or RPMI-1640, respectively, 50 U/ml penicillin, and 100 μg/ml streptomycin. These cells were incubated at 37°C in a humidified atmosphere of 5% CO_2_.

### Reagents

Resibufogenin was purchased from Matsuura Yakugyo. MG132 was purchased from Peptide Institute. SB216763 was purchased from Sigma-Aldrich. These reagents were dissolved in DMSO.

### Cell viability assay

After the incubation of cells for 1, 2, or 3 days with the indicated concentrations of resibufogenin, the number of viable cells and dead cells was measured by a Guava EasyCyte plus flow cytometer according to the manufacturer’s instructions (Millipore).

### Determination of apoptosis by annexin V staining

Cells were treated with resibufogenin at the indicated concentrations or celecoxib, a positive control as an apoptosis-inducer, for 24 h. Subsequently, cells were subjected to annexin V staining using the Vybrant Apoptosis Assay Kit (Molecular Probes) according to manufacturer’s instructions. The stained cells were measured by FACSCalibur (Becton Dickinson).

### Cell cycle analysis

Cells were incubated with the indicated concentrations of resibufogenin for 24 h. The cells were then fixed in 0.2% Triton X-100 (Nacalai Tesque), treated with 300 μg/ml RNase A (Sigma-Aldrich), and their nuclei were stained with 10 μg/ml propidium iodide (Sigma-Aldrich). The stained nuclei were measured by FACSCalibur. The data was analysed using Modfit LT software (Verity Software House).

### 5-Bromo-2’-deoxyuridine (BrdU) incorporation

Cells were treated with resibufogenin at the indicated concentrations for 24 h. Subsequently, the cells were incubated with BrdU for 2 h. The incorporation of BrdU into DNA was measured using a cell proliferation enzyme-linked immunosorbent BrdU assay kit (Roche).

### Protein isolation and Western blot analysis

Cells were lysed in buffer containing 50 mM Tris-HCl (pH 7.5), 1% SDS, 1 mM DTT, 0.43 mM 4-(2-aminoethyl) benzenesulfonyl fluoride hydrochloride, and Phosphatase Inhibitor Cocktail (Nacalai Tesque). The lysate was sonicated, centrifuged, and the supernatant was collected. The protein extract was boiled with sample buffer for 5 min, subjected to SDS-PAGE, and transferred to PVDF membrane (Millipore). Anti-human phospho-RB (Ser780) and phospho-RB (Ser807/811) (Cell Signaling Technology) rabbit antibodies, and anti-human cyclin D1 (MBL), RB (BD Pharmingen) and GAPDH (HyTest) mouse antibodies were used as the primary antibodies. The signals were developed with Chemi-Lumi One L (Nacalai Tesque) or Immobilon Western (Millipore). The relative band intensity against each loading control was assessed by densitometric analysis using ImageJ software [20].

### Statistical analysis

Statistical analysis of the data was performed using the Student’s *t*-test for comparison between treatments and controls. *P* < 0.05 was considered significant.

## Results

### Resibufogenin inhibits the growth of human colon cancer HT-29 cells

**Fig 2:**
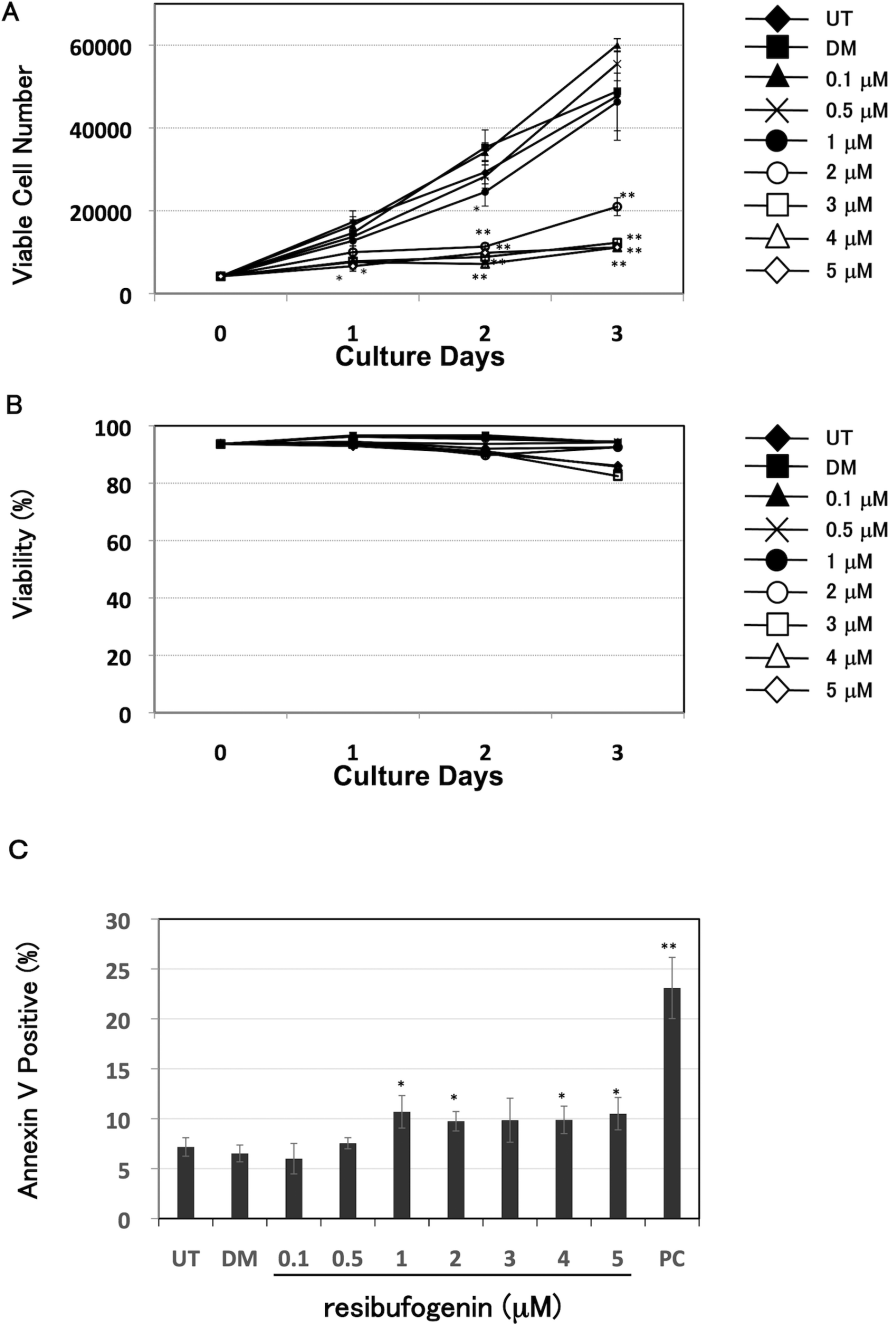
Resibufogenin inhibits the growth of human colon cancer HT-29 cells. (A) Growth inhibitory effect of resibufogenin by counting viable cell number. Cells were treated with resibufogenin at the indicated concentrations for 1, 2, or 3 days. Viable cell number was measured by a Guava EasyCyte plus flow cytometry. UT, untreated; DM, treated with DMSO. Points, means (n = 3); bars, SD. *P < 0.05, **P < 0.01, compared with the DMSO-treated control. (B) The effect of resibufogenin on cell viability. Cells were treated and measured as shown in (A). Viability was calculated using the number of viable cells and dead cells. UT, untreated; DM, treated with DMSO. Points, means (n = 3) (C) The effect of resibufogenin on apoptosis by annexin V staining. Cells were treated with resibufogenin at the indicated concentrations for 24 h, and subjected to annexin V staining. The stained cells were measured by FACSCalibur. UT, untreated; DM, treated with DMSO. Celecoxib at 100 μM was used as a positive control (PC). Points, means (n = 3); bars, SD. *P < 0.05, **P < 0.01, compared with the DMSO-treated control.

We investigated the effect of resibufogenin on the growth of HT-29 cells using a Guava EasyCyte plus flow cytometer. Resibufogenin at 2 μM or more for 2 and 3 days significantly (*P* < 0.01) inhibited the viable cell number (Fig 2A). Next, we investigated whether resibufogenin induced cell death using a Guava EasyCyte plus flow cytometer. As shown in Fig 2B, viability was not drastically changed. Furthermore, we investigated whether resibufogenin induced apoptosis using annexin V staining. As shown in Fig 2C, resibufogenin slightly induced apoptosis. However, the induction of apoptosis by resibufogenin was much weaker than that by celecoxib, which is known to induce apoptosis in HT-29 cells [21]. These results suggest that resibufogenin inhibits the growth of HT-29 cells partially through slight induction of apoptosis.

### Resibufogenin induces G1-phase arrest in HT-29 cells

**Fig 3:**
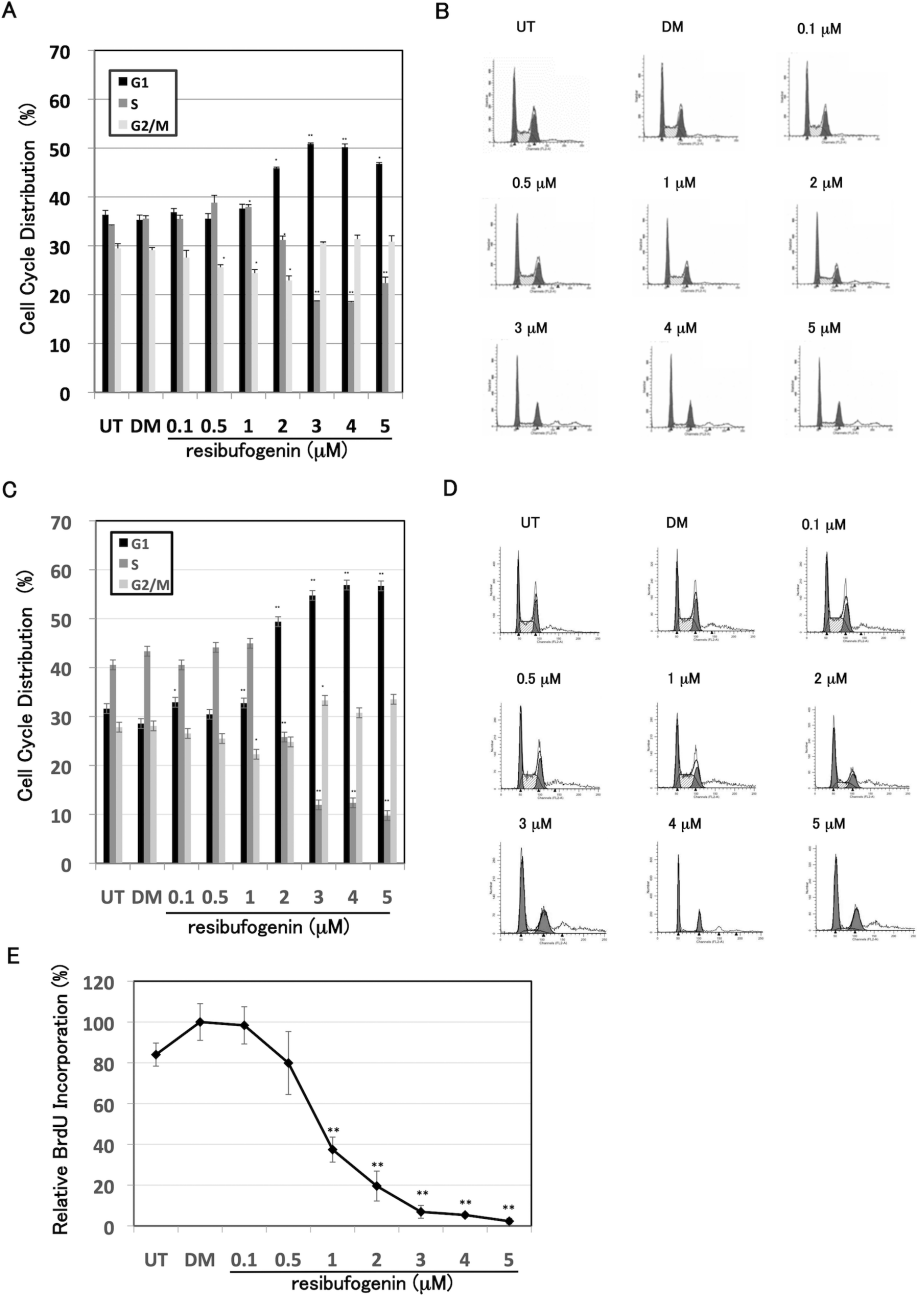
Resibufogenin induces G1-phase arrest in HT-29 cells. (A) Cell cycle analysis of HT-29 cells treated with resibufogenin. Cells were treated with resibufogenin at the indicated concentrations for 24 h. The DNA content of propidium iodide-stained nuclei was analysed by flow cytometry. The percentages in G1 (black), S (dark gray) and G2/M (light gray) phases of the cell cycle were analysed using Modfit LT software. UT, untreated; DM, treated with DMSO. Points, means (n = 3); bars, SD. *P < 0.05, **P < 0.01, compared with the DMSO-treated control. (B) Representative histogram of each treatment. (C) Cell cycle analysis of HT-29 cells treated with resibufogenin in synchronized cells by serum starvation. Cells were treated with resibufogenin at the indicated concentrations for 24 h after the 24 h serum starvation without FBS. The cell cycle analysis was similarly performed and shown as described in the figure legend of Fig 3A above. (D) Representative histogram of each treatment. (E) BrdU incorporation analysis of HT-29 cells treated with resibufogenin. Cells were treated with resibufogenin at the indicated concentrations for 24 h. Subsequently, the cells were incubated with BrdU for 2 h. The incorporation of BrdU into DNA was measured using a cell proliferation enzyme-linked immunosorbent BrdU assay kit. The data obtained with DMSO was taken as 100%. UT, untreated; DM, treated with DMSO. Points, means (n = 3); bars, SD. **P < 0.01, compared with the DMSO-treated control.

We performed a cell cycle analysis using flow cytometry. Resibufogenin treatment at 2 μM or more significantly (*P* < 0.05) increased the G1-phase with a decrease in the S-phase (Fig 3A). Representative histogram in each sample is shown in Fig 3B. We also performed a cell cycle analysis in synchronized cells by serum starvation. Resibufogenin treatment at 2 μM or more drastically increased the G1-phase in synchronized cells, and the increase of G1-phase in synchronized cells was more apparent than that in unsynchronized cells (Fig 3C). Representative histogram in each sample is shown in Fig 3D. Furthermore, we also examined the effect of resibufogenin on BrdU incorporation. Resibufogenin treatment at 1 μM or more significantly suppressed BrdU incorporation (Fig 3E). These results indicate that resibufogenin arrests the cell cycle at G1-phase and inhibits the entry to S-phase. Since resibufogenin induced G1-phase arrest, we performed β-galactosidase assay to investigate whether resibufogenin induced senescence or not. As a result, resibufogenin treatment did not induce SA-β-Gal activity (S1 Fig). These results demonstrate that resibufogenin induces G1-phase arrest but not senescence.

### Resibufogenin converts RB protein to the unphosphorylated form with down-regulation of cyclin D1 expression

**Fig 4:**
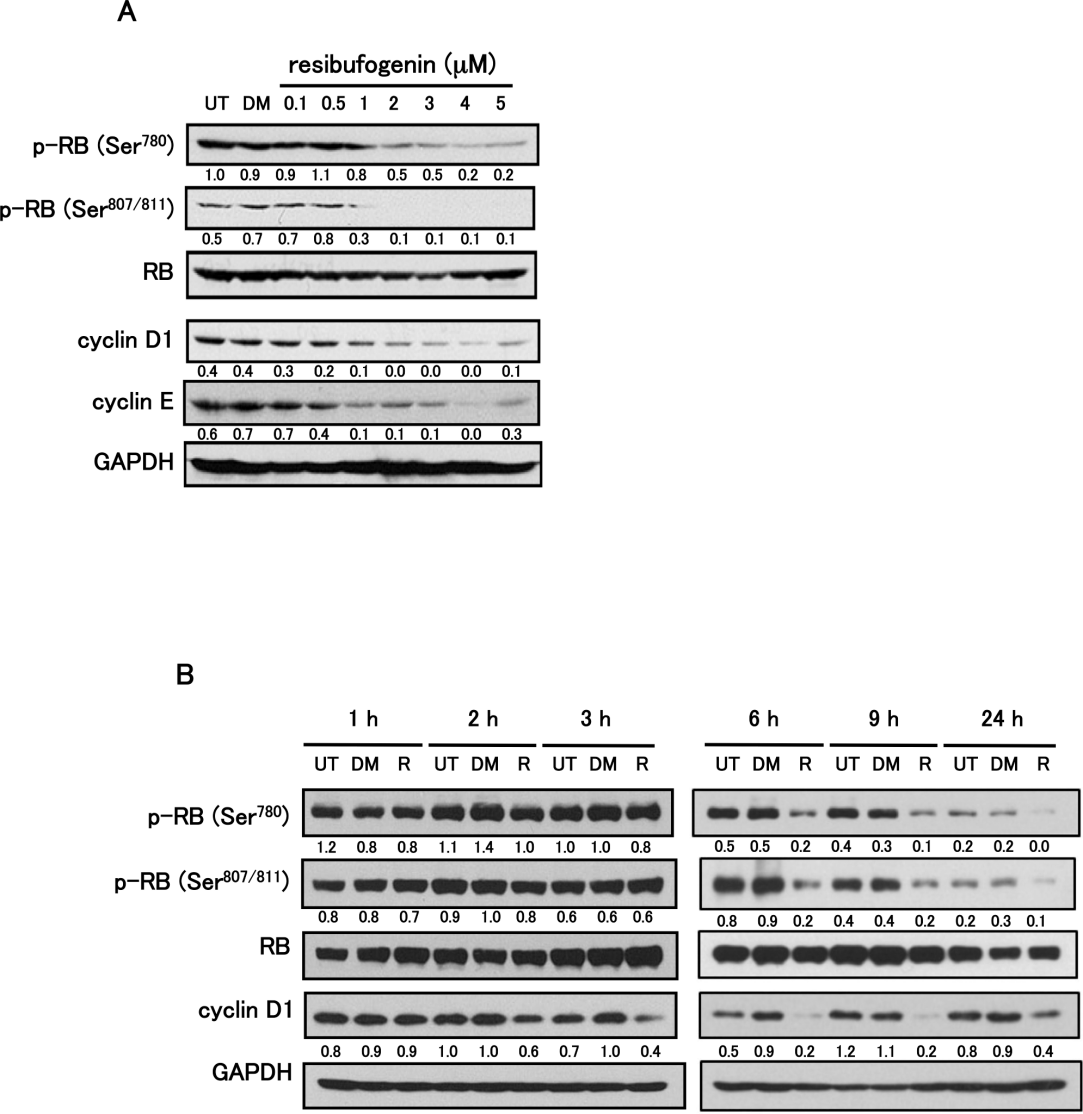
Resibufogenin converts RB protein to the unphosphorylated form with down-regulation of cyclin D1 expression. (A) The effect of resibufogenin on the expression of phosphorylated RB (p-RB (Ser^780^) and p-RB (Ser^807/811^)), RB, cyclin D1 and cyclin E. Cells were treated with resibufogenin at the indicated concentrations for 24 h and analysed by Western blotting. (B) Time-course study on the phosphorylation status of RB protein and the expression of cyclin D1. Cells were treated with 5 μM resibufogenin (R) for 1, 2, 3, 6, 9 or 24 h and analysed by Western blotting. In both figures, RB or GAPDH was used as a loading control for protein quantitation. UT, untreated; DM, treated with DMSO. The band intensity was measured and normalized by RB or GAPDH, and the protein levels are shown at the bottom of each blot.

We investigated whether resibufogenin could convert RB protein to the unphosphorylated form with down-regulation of cyclin D1 and/or cyclin E expressions. Resibufogenin at 1 μM or more decreased phosphorylated form of RB with down-regulation of cyclin D1 and cyclin E expressions in HT-29 cells (Fig 4A). On the other hand, resibufogenin did not dose-dependently change the expressions of CDK2, CDK4, CDK6, p15^INK4b^, p16^INK4a^, p18^INK4c^, p19^INK4d^, p21^WAF1/Cip1^ and p27^Kip1^ (S2 Fig). We further performed the time-course study of the phosphorylation status of RB protein and the expression of cyclin D1. Three hours after the treatment, resibufogenin down-regulated cyclin D1 expression, and 6 h after the treatment resibufogenin reduced the phosphorylated form of RB protein in HT-29 cells (Fig 4B). Almost similar results were obtained in human lung cancer A549 cells (S3 Fig). It raises the possibility that conversion of RB protein to the unphosphorylated form may be caused by the preceding reduction of cyclin D1.

### Resibufogenin down-regulates cyclin D1 through proteasomal degradation

**Fig 5:**
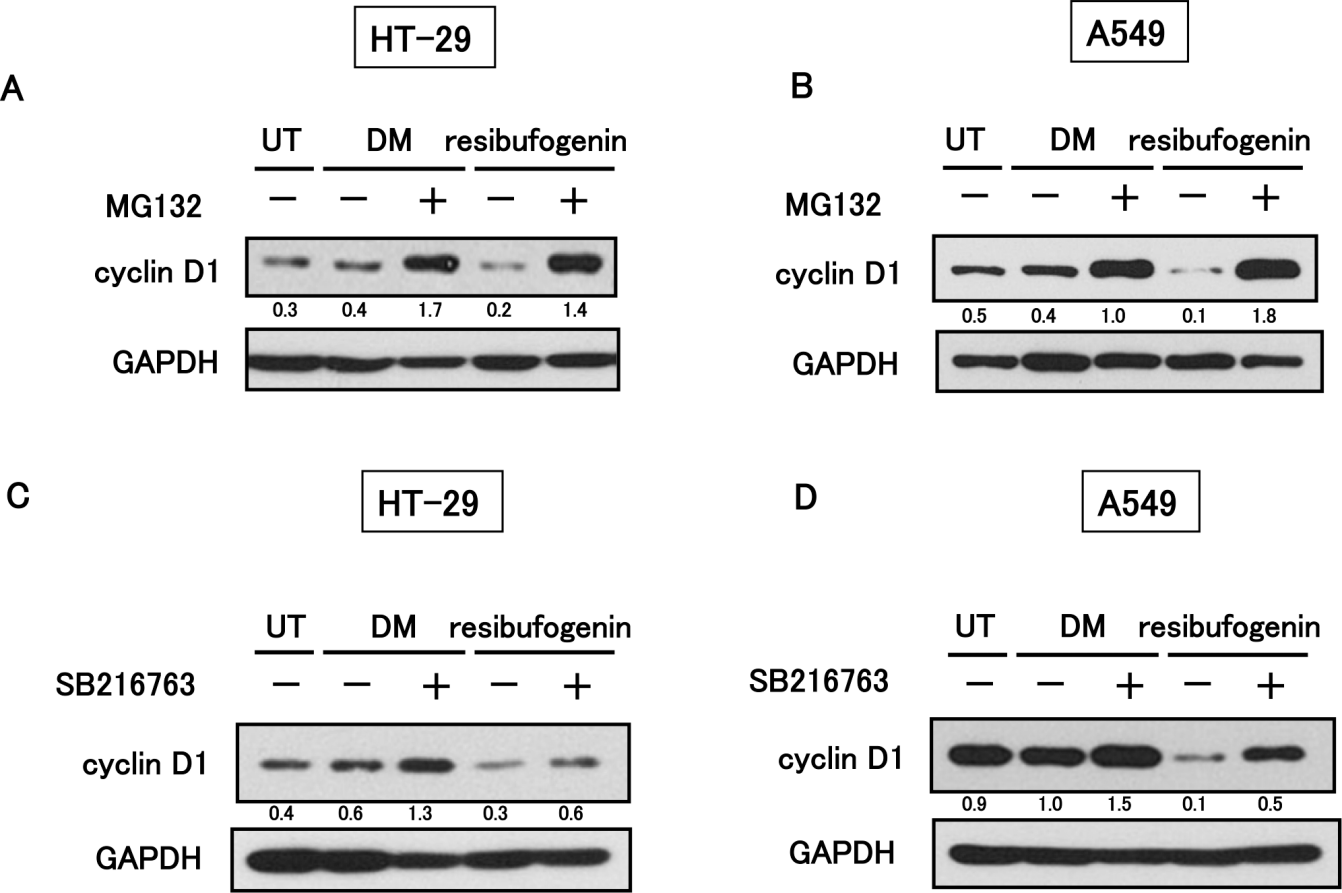
Resibufogenin down-regulates cyclin D1 through proteasomal degradation. (A) The effect of MG132 on suppression of cyclin D1 expression by resibufogenin in HT-29 cells. Cells were treated by 5 μM resibufogenin with or without 20 μM MG132 for 24 h. (B) The effect of MG132 on suppression of cyclin D1 expression by resibufogenin in A549 cells. Cells were treated with 2.5 μM resibufogenin with or without 20 μM MG132 for 24 h. (C) The effect of SB216763 on suppression of cyclin D1 expression by resibufogenin in HT-29 cells. Cells were treated by 5 μM resibufogenin with or without 30 μM SB216763 for 3 h. (D) The effect of SB216763 on suppression of cyclin D1 expression by resibufogenin in A549 cells. Cells were treated with 2.5 μM resibufogenin with or without 30 μM SB216763 for 6 h. In all figures, GAPDH was used as a loading control for protein quantitation. UT, untreated; DM, treated with DMSO. The band intensity was measured and normalized by GAPDH, and the protein levels are shown at the bottom of each blot.

To elucidate the molecular mechanism of reduction of cyclin D1 induced by resibufogenin, we first investigated the effect of resibufogenin on cyclin D1 mRNA expression using quantitative RT-PCR. Resibufogenin did not decrease but increased the cyclin D1 mRNA level (S4 Fig), suggesting the importance of the regulation at a protein level. Therefore, we examined the stability of cyclin D1 when treated with resibufogenin. HT-29 and A549 cells were treated by resibufogenin with or without MG132, a proteasome inhibitor, and then analysed for cyclin D1 expression using Western blotting. MG132 completely recovered the resibufogenin-induced reduction of cyclin D1 (Fig 5A and 5B). These results indicate that resibufogenin down-regulates cyclin D1 through the proteasomal degradation.

### GSK-3β is required for resibufogenin-induced cyclin D1 degradation

It has been reported that GSK-3β phosphorylates cyclin D1 at Thr^286^ and causes subsequent proteasomal degradation [17–19]. To elucidate the role of GSK-3β in resibufogenin-mediated cyclin D1 degradation, we used SB216763, a specific inhibitor of GSK-3β. HT-29 and A549 cells were treated by resibufogenin with or without SB216763. SB216763 attenuated the degradation of cyclin D1 by resibufogenin (Fig 5C and 5D). These results suggest that resibufogenin induces the cyclin D1 degradation through GSK-3β. Since β-catenin is a well-known substrate of GSK-3β and degraded by subsequent proteasomal degradation [22], we examined the expression level of β-catenin. Resibufogenin also down-regulated β-catenin expression similarly to cyclin D1 in HT-29 cells (S5 Fig). These results suggest resibufogenin might activate GSK-3β, resulting in the down-regulation of both cyclin D1 and β-catenin.

## Discussion

In this study, we found that resibufogenin induced G1-phase arrest with the conversion of RB protein to the unphosphorylated form with down-regulation of cyclin D1 protein through the proteasomal degradation in human colon cancer HT-29 cells and human lung cancer A549 cells. On the other hand, cyclin D1-CDK4/6 complex phosphorylates and inactivates RB protein resulting in promoting the G1/S phase transition [10]. Interestingly, cyclin D1 is frequently over-expressed in human malignant tumors [12–14, 23]. For example, cyclin D1 gene is over-expressed in 40% of human breast cancers [24], and high cyclin D1 expression levels correlate with the low survival probability for patients with lung cancer [13]. In the present study, resibufogenin down-regulated cyclin D1 through the proteasomal degradation (Fig 5A and 5B), thereby suggesting that resibufogenin might be useful for the treatment of malignant tumors with over-expression of cyclin D1. Furthermore, in cancer cell lines with mutations in both KRAS and PIK3CA, MEK inhibitor could not induce the cell cycle arrest due to the sustained cyclin D1 expression [25]. This suggests that cyclin D1 expression is correlated with the resistance of MEK inhibitor. Therefore, the down-regulation of cyclin D1 by resibufogenin may be also useful to overcome the resistance of MEK inhibitor in malignant tumors with mutations in both KRAS and PIK3CA.

Cyclin D1 in the nucleus is phosphorylated by GSK-3β and subsequently exported to cytoplasm [18, 19]. Phosphorylated cyclin D1 in cytoplasm is ubiquitinated by SCF^FBX4-αB Crystallin^ and subsequently degraded by 26S proteasome [16]. GSK-3β is phosphorylated and inactivated by Akt and p38 MAPK [26, 27]. Ser^9^ of GSK-3β is phosphorylated by Akt and Thr^390^ is phosphorylated by p38 MAPK, respectively. Although we found that resibufogenin induced the cyclin D1 degradation depending on the activity of GSK-3β in this study, resibufogenin did not decrease the phosphorylation status of GSK-3β (S6 Fig). Our results suggest that resibufogenin might activate GSK-3β through the Akt- and p38 MAPK-independent pathway.

Recently, huachansu, which is dried venom secreted from the skin glands of *Bufo bufo gargarizans* Cantor, has been expected as a candidate of anticancer agents [28]. Its anticancer potential was considered as an inhibitor of NF-κB [28]. We demonstrated here, for the first time, that resibufogenin, which is a component of huachansu, a traditional Chinese medicine, induced the cyclin D1 degradation through the activation of GSK-3β. Therefore, resibufogenin might be worth developing as a possible treatment for the malignant tumors with cyclin D1 over-expression.

## Supporting Information

S1 Fig Resibufogenin dose not induce senescence. After the treatment of DMSO or 5 μM resibufogenin (R) for 24 h, SA-β-gal activity was measured using Cellular Senescence Assay Kit (Cell Biolabs). The obtained data were normalized to protein concentrations. UT, untreated; DM, treated with DMSO. Points, means (n = 3); bars, SD.
(TIFF)

S2 Fig Resibufogenin does not dose-dependently change the expressions of CDK2, CDK4, CDK6, p15^INK4b^, p16^INK4a^, p18^INK4c^, p19^INK4d^, p21^WAF1/Cip1^ and p27^Kip1^. Cells were treated with resibufogenin at the indicated concentrations for 24 h and analysed by Western blotting. GAPDH was used as a loading control for protein quantitation. UT, untreated; DM, treated with DMSO. The band intensity was measured and normalized by GAPDH, and the protein levels are shown at the bottom of each blot. The image of GAPDH for CDK2, CDK6 and p19^INK4d^ is the same as that in Fig 4A due to the same gel.
(TIFF)

S3 Fig Resibufogenin converts RB protein to the unphosphorylated form with down-regulation of cyclin D1 expression in A549 cells. (A) The effect of resibufogenin on the expression of phosphorylated RB (p-RB (Ser^780^) and p-RB (Ser^807/811^)) and cyclin D1. A549 cells were treated with resibufogenin at the indicated concentrations for 24 h and analysed by Western blotting. (B) Time-course study on the phosphorylation status of RB protein and the expression of cyclin D1. A549 cells were treated with 2.5 μM resibufogenin (R) for 3, 6, or 9 h and analysed by Western blotting. In both figures, RB or GAPDH was used as a loading control for protein quantitation. UT, untreated; DM, treated with DMSO. The band intensity was measured and normalized by RB or GAPDH, and the protein levels are shown at the bottom of each blot.
(TIFF)

S4 Fig Resibufogenin does not decrease but increases cyclin D1 mRNA expression in HT-29 cells. Cells were treated with resibufogenin at the indicated concentrations for 24 h. Cyclin D1 mRNA was measured by quantitative RT-PCR. Cyclin D1 mRNA was normalized to GAPDH mRNA, and the data obtained with DMSO was taken as 1. UT, untreated; DM, treated with DMSO. Data are shown as means (n = 3) ± SD. *P < 0.05, **P < 0.01, compared with the DMSO-treated control.
(TIFF)

S5 Fig Resibufogenin down-regulates the expression of β-catenin. HT-29 cells were treated by 5 μM resibufogenin (R) for 9 h. The expression of β-catenin was analysed by Western blotting. GAPDH was used as a loading control for protein quantitation. UT, untreated; DM, treated with DMSO. The band intensity was measured and normalized by GAPDH, and the protein levels are shown at the bottom of each blot.
(TIFF)

S6 Fig Resibufogenin does not decrease the phosphorylation status of GSK-3β. The effect of resibufogenin on the phosphorylation status of GSK-3β (Ser^9^) (A) or GSK-3β (Thr^390^) (C) in HT-29 cells. Cells were treated by 5 μM resibufogenin (R) for 1, 2, or 3 h, and analysed by Western blotting. The effect of resibufogenin on the phosphorylation status of GSK-3β (Ser^9^) (B) or GSK-3β (Thr^390^) (D) in A549 cells. Cells were treated by 2.5 μM resibufogenin (R) for 3, 6, or 9 h, and analysed by Western blotting. In all figures, GSK-3β was used as a loading control for protein quantitation. UT, untreated; DM, treated with DMSO. The band intensity was measured and normalized by GSK-3β, and the protein levels are shown at the bottom of each blot.
(TIFF)

S1 Materials and Methods Supporting materials and methods. (DOCX)
