# Supplementary figures and images for: Resibufogenin Induces G1-Phase Arrest through the Proteasomal Degradation of Cyclin D1 in Human Malignant Tumor Cells
Source: PLoS One. 2015 Jun 29;10(6):e0129851. doi: 10.1371/journal.pone.0129851 (PMC4488249; doi:10.1371/journal.pone.0129851)

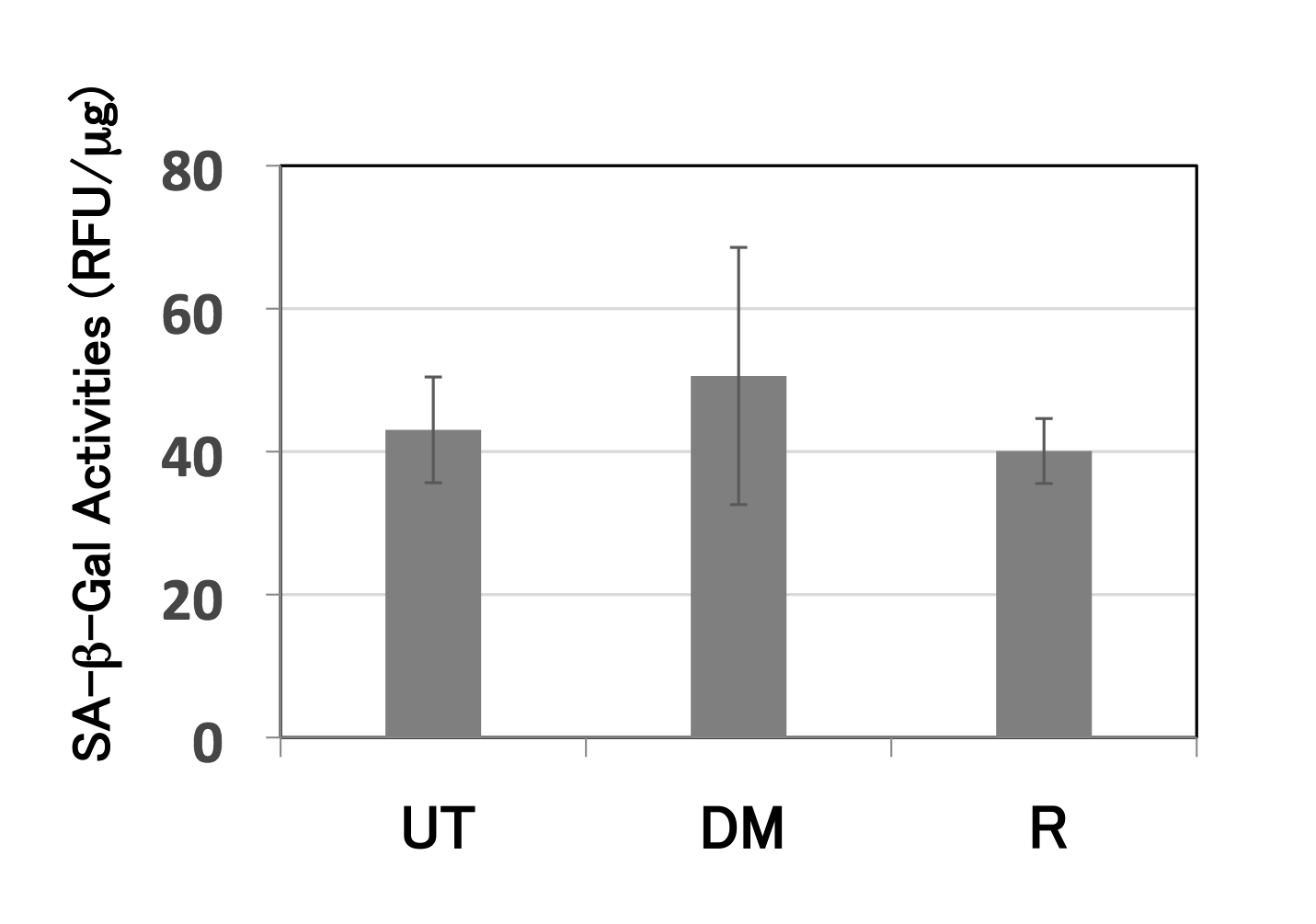

Supplement: S1 Fig — After the treatment of DMSO or 5 μM resibufogenin (R) for 24 h, SA-β-gal activity was measured using Cellular Senescence Assay Kit (Cell Biolabs). The obtained data were normalized to protein concentrations. UT, untreated; DM, treated with DMSO. Points, means (n = 3); bars, SD. (TIFF) [file pone.0129851.s001.tiff]

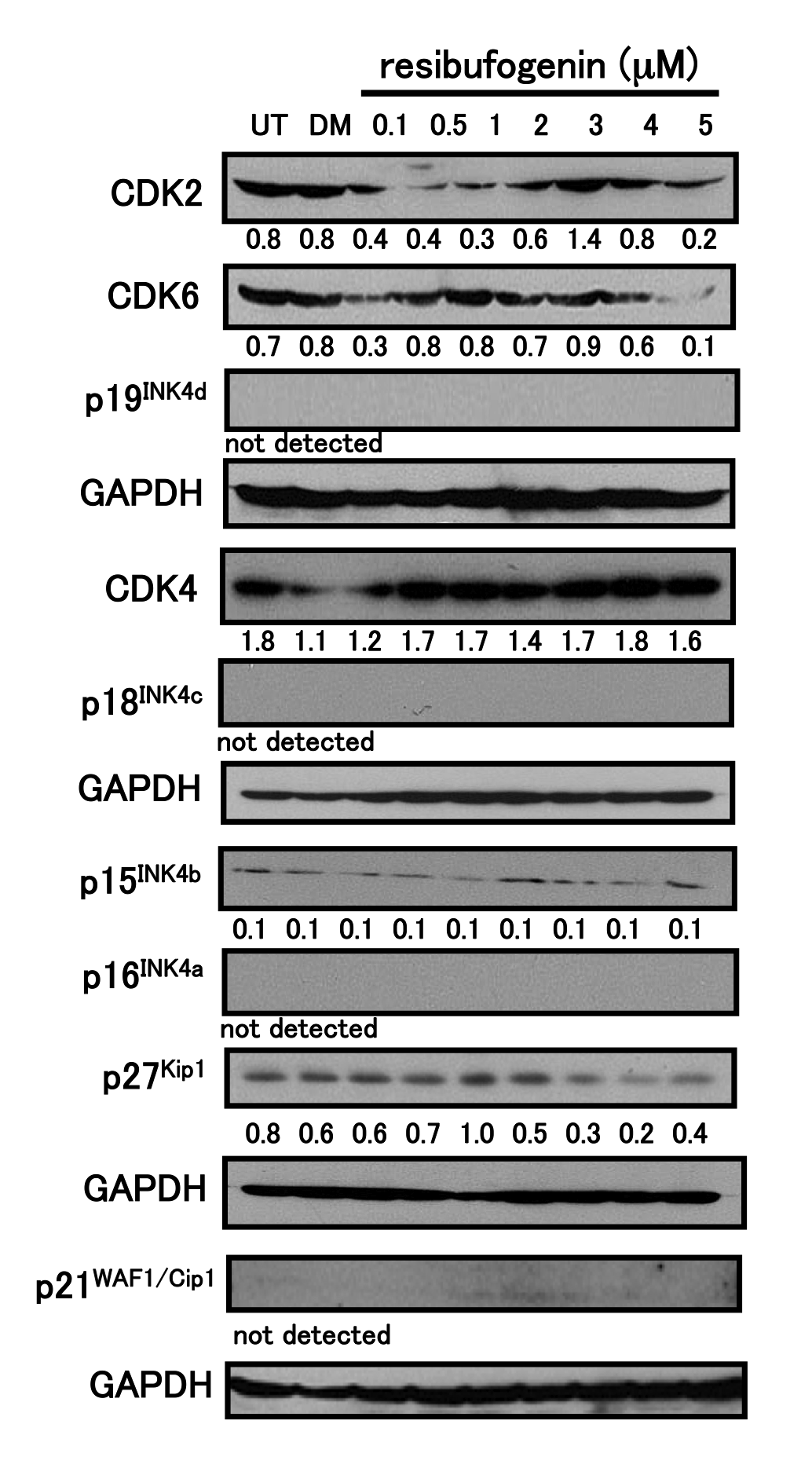

Supplement: S2 Fig — Cells were treated with resibufogenin at the indicated concentrations for 24 h and analysed by Western blotting. GAPDH was used as a loading control for protein quantitation. UT, untreated; DM, treated with DMSO. The band intensity was measured and normalized by GAPDH, and the protein levels are shown at the bottom of each blot. The image of GAPDH for CDK2, CDK6 and p19INK4d is the same as that in Fig 4A due to the same gel. (TIFF) [file pone.0129851.s002.tiff]

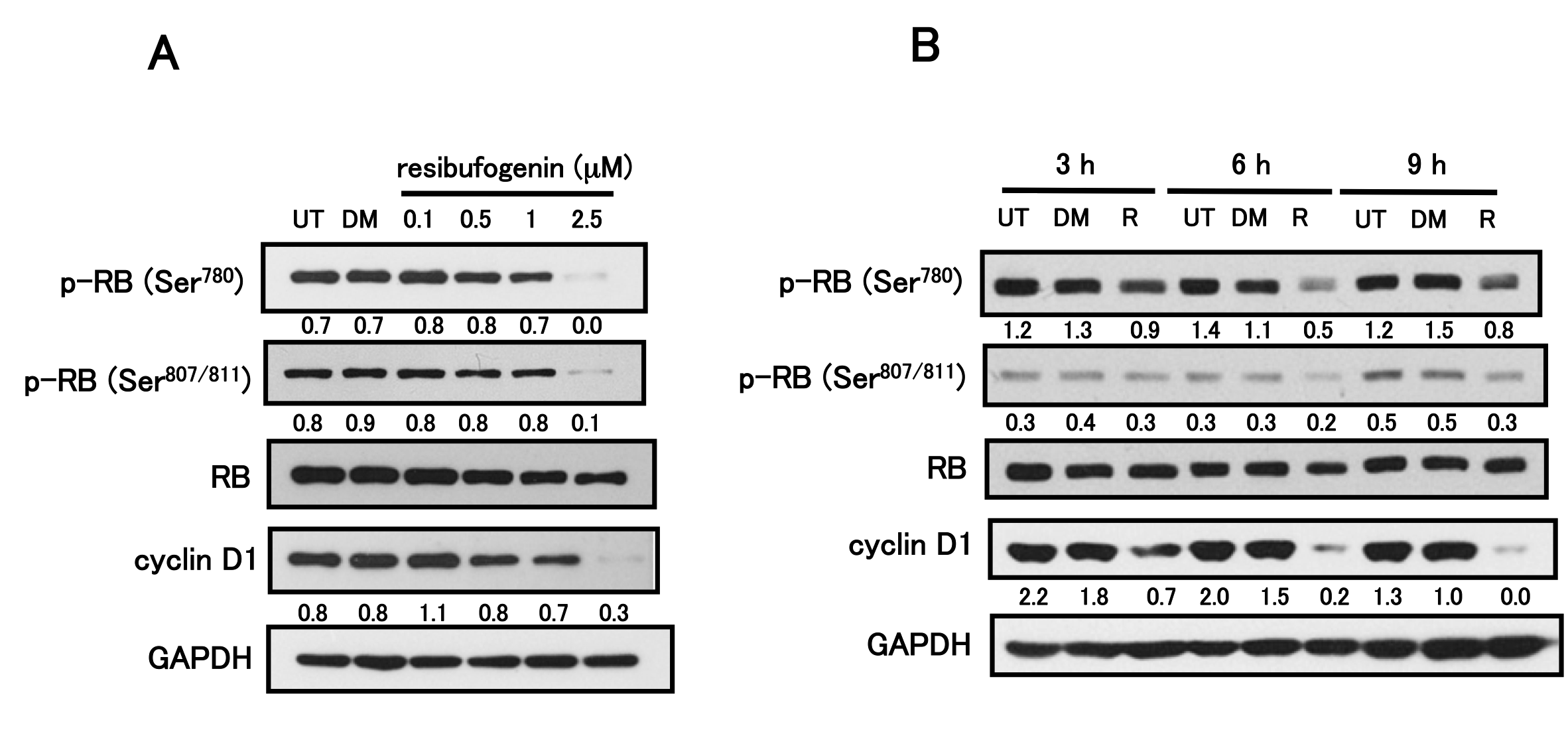

Supplement: S3 Fig — (A) The effect of resibufogenin on the expression of phosphorylated RB (p-RB (Ser780) and p-RB (Ser807/811)) and cyclin D1. A549 cells were treated with resibufogenin at the indicated concentrations for 24 h and analysed by Western blotting. (B) Time-course study on the phosphorylation status of RB protein and the expression of cyclin D1. A549 cells were treated with 2.5 μM resibufogenin (R) for 3, 6, or 9 h and analysed by Western blotting. In both figures, RB or GAPDH was used as a loading control for protein quantitation. UT, untreated; DM, treated with DMSO. The band intensity was measured and normalized by RB or GAPDH, and the protein levels are shown at the bottom of each blot. (TIFF) [file pone.0129851.s003.tiff]

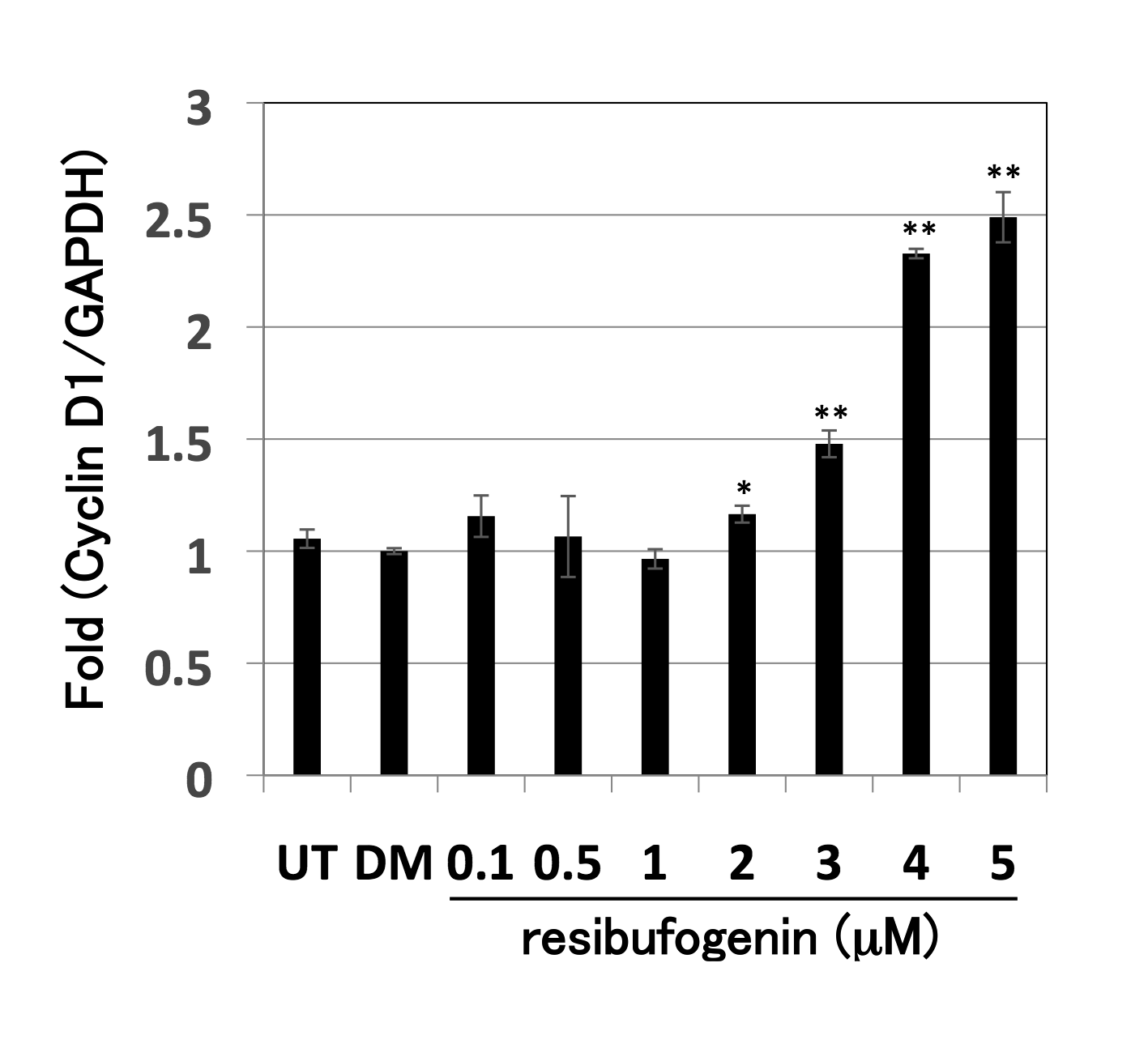

Supplement: S4 Fig — Cells were treated with resibufogenin at the indicated concentrations for 24 h. Cyclin D1 mRNA was measured by quantitative RT-PCR. Cyclin D1 mRNA was normalized to GAPDH mRNA, and the data obtained with DMSO was taken as 1. UT, untreated; DM, treated with DMSO. Data are shown as means (n = 3) ± SD. *P < 0.05, **P < 0.01, compared with the DMSO-treated control. (TIFF) [file pone.0129851.s004.tiff]

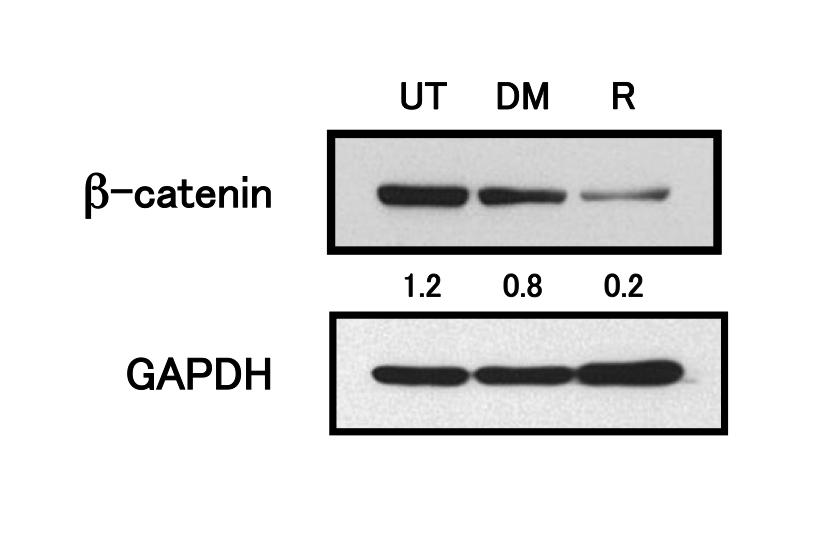

Supplement: S5 Fig — HT-29 cells were treated by 5 μM resibufogenin (R) for 9 h. The expression of β-catenin was analysed by Western blotting. GAPDH was used as a loading control for protein quantitation. UT, untreated; DM, treated with DMSO. The band intensity was measured and normalized by GAPDH, and the protein levels are shown at the bottom of each blot. (TIFF) [file pone.0129851.s005.tiff]

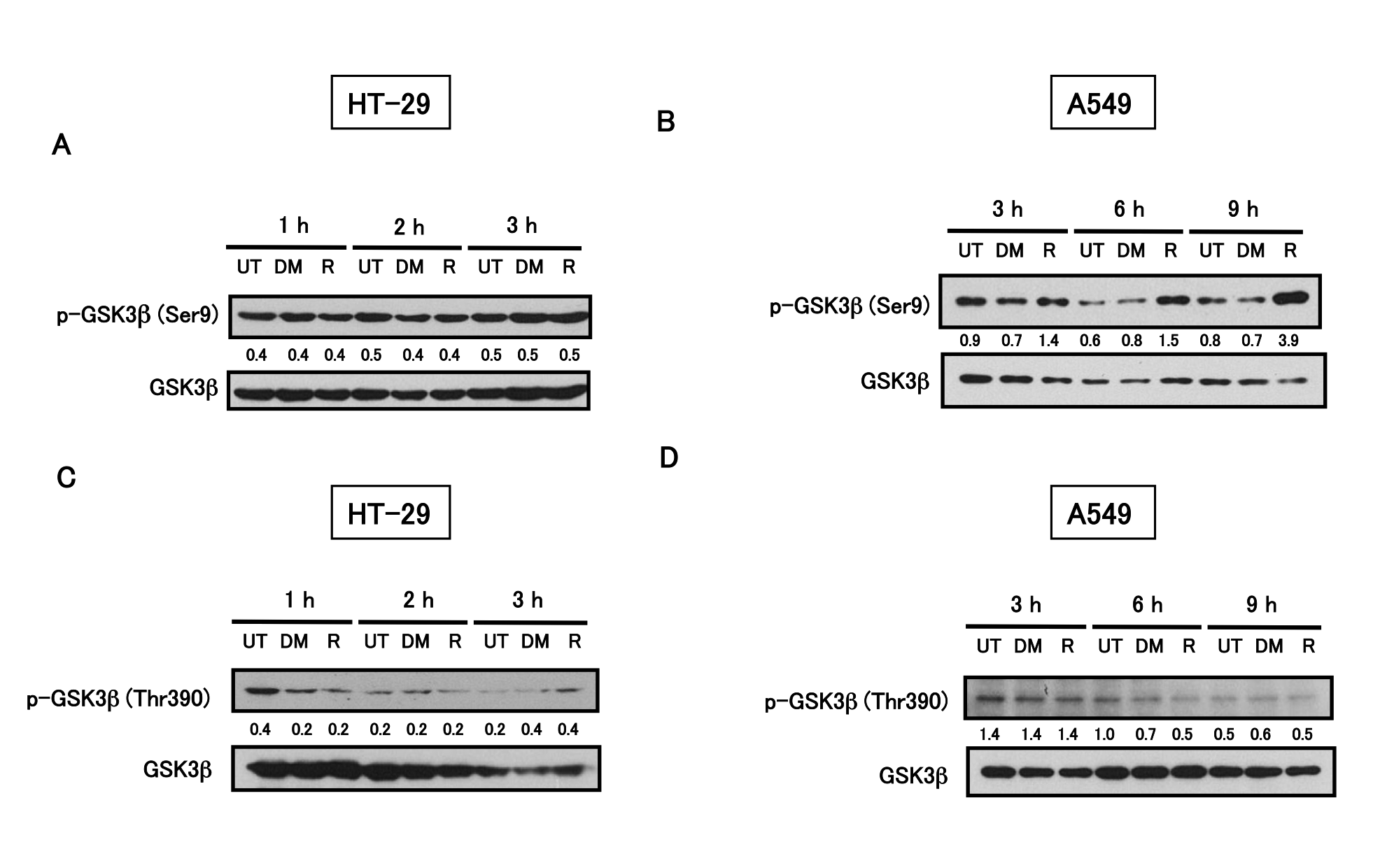

Supplement: S6 Fig — The effect of resibufogenin on the phosphorylation status of GSK-3β (Ser9) (A) or GSK-3β (Thr390) (C) in HT-29 cells. Cells were treated by 5 μM resibufogenin (R) for 1, 2, or 3 h, and analysed by Western blotting. The effect of resibufogenin on the phosphorylation status of GSK-3β (Ser9) (B) or GSK-3β (Thr390) (D) in A549 cells. Cells were treated by 2.5 μM resibufogenin (R) for 3, 6, or 9 h, and analysed by Western blotting. In all figures, GSK-3β was used as a loading control for protein quantitation. UT, untreated; DM, treated with DMSO. The band intensity was measured and normalized by GSK-3β, and the protein levels are shown at the bottom of each blot. (TIFF) [file pone.0129851.s006.tiff]
