# Supplementary material for: Resibufogenin Induces G1-Phase Arrest through the Proteasomal Degradation of Cyclin D1 in Human Malignant Tumor Cells
Source: PLoS One. 2015 Jun 29;10(6):e0129851. doi: 10.1371/journal.pone.0129851 (PMC4488249; doi:10.1371/journal.pone.0129851)
Supplement: S1 Materials and Methods — (DOCX) [file pone.0129851.s007.docx]

**Supporting information**

**Materials and methods**

**Reagents**

Anti-human CDK2, CDK4, p15^INK4b^, p16^INK4a^, p19^INK4d^, p21^WAF1/Cip1^, p27^Kip1^ (Santa Cruz Biotechnology), β-catenin, phospho-GSK-3β (Ser9), phospho-GSK-3β (Thr390) and GSK-3β (Cell Signaling Technology) rabbit antibodies, and anti-human CDK6 (Cell Signaling Technology) mouse antibody were used as the primary antibodies.

**Quantitative RT-PCR**

Total RNA was isolated from cells treated with resibufogenin at the indicated concentrations for 24 h using Sepasol-RNA I super (Nacalai Tesque) according to the manufacturer’s instructions. cDNA was synthesized from total RNA using High-Capacity cDNA Reverse Transcription Kits (Applied Biosystems). Quantitative RT-PCR was performed using TaqMan Probes (Applied Biosystems) and an ABI 7300 RT-PCR system (Applied Biosystems).
